# Hydrodynamic disturbance controls microbial community assembly and biogeochemical processes in coastal sediments

**DOI:** 10.1038/s41396-021-01111-9

**Published:** 2021-09-28

**Authors:** Ya-Jou Chen, Pok Man Leung, Perran L. M. Cook, Wei Wen Wong, Tess Hutchinson, Vera Eate, Adam J. Kessler, Chris Greening

**Affiliations:** 1 Department of Microbiology, Biomedicine Discovery Institute, Clayton, VIC 3800 Australia; 2 School of Biological Sciences, Monash University, Clayton, VIC 3800 Australia; 3 Water Studies Centre, School of Chemistry, Monash University, Clayton, VIC 3800 Australia; 4 School of Earth, Atmosphere and Environment, Monash University, Clayton, VIC 3800 Australia

**Keywords:** Water microbiology, Biogeochemistry

## Abstract

The microbial community composition and biogeochemical dynamics of coastal permeable (sand) sediments differs from cohesive (mud) sediments. Tide- and wave-driven hydrodynamic disturbance causes spatiotemporal variations in oxygen levels, which select for microbial generalists and disrupt redox cascades. In this work, we profiled microbial communities and biogeochemical dynamics in sediment profiles from three sites varying in their exposure to hydrodynamic disturbance. Strong variations in sediment geochemistry, biogeochemical activities, and microbial abundance, composition, and capabilities were observed between the sites. Most of these variations, except for microbial abundance and diversity, significantly correlated with the relative disturbance level of each sample. In line with previous findings, metabolically flexible habitat generalists (e.g., Flavobacteriaceae, Woeseaiceae, Rhodobacteraceae) dominated in all samples. However, we present evidence that aerobic specialists such as ammonia-oxidizing archaea (Nitrosopumilaceae) were more abundant and active in more disturbed samples, whereas bacteria capable of sulfate reduction (e.g., uncultured Desulfobacterales), dissimilatory nitrate reduction to ammonium (DNRA; e.g., Ignavibacteriaceae), and sulfide-dependent chemolithoautotrophy (e.g., Sulfurovaceae) were enriched and active in less disturbed samples. These findings are supported by insights from nine deeply sequenced metagenomes and 169 derived metagenome-assembled genomes. Altogether, these findings suggest that hydrodynamic disturbance is a critical factor controlling microbial community assembly and biogeochemical processes in coastal sediments. Moreover, they strengthen our understanding of the relationships between microbial composition and biogeochemical processes in these unique environments.

## Introduction

The defining feature of intertidal sediments is their exposure to regular hydrodynamic disturbance due to tidal flows and waves. The resultant frequent variations in physicochemical conditions exert major selective pressures on the microorganisms that control biogeochemical cycling in these environments [1, 2]. The low permeability of cohesive (mud/silt) sediments buffers microorganisms from disturbance; as a result, these systems become depth-stratified in redox state, community composition, and biogeochemical reactions [3]. The scenario is very different for the permeable (sand/gravel) sediments that span half of continental shelves [4, 5]. Pressure gradients form at these sites due to interaction of wave action with sediment topography, bottom currents, and bioirrigation. These gradients force water to flow through sediment through the process of advective transport [2, 6, 7], resulting in rapid exchange of dissolved particles, solutes, gases, and microorganisms between porewater and sediment grains [8–10]. In turn, these physical processes cause large variations in the levels of hydration, oxygen, light, and nutrients available to grain-associated microorganisms across short spatial and temporal scales [2, 11, 12]. Various factors, including the degree of tide- and wave-driven hydrodynamic force on sediments, control the extent of porewater advection and in turn the spatiotemporal variability of these systems [13, 14]. This disturbance is predicted to profoundly influence microbial community assembly and biogeochemical processes.

Permeable sediments host microbial communities that are distinct from those of cohesive sediments [15–20]. Variations in resource availability and oxygen exposure select for flexible habitat generalists rather than niche-restricted specialists [21]. Consistently, many of the most abundant and prevalent bacterial lineages in permeable sediments, most notably Woeseiaceae and Flavobacteriaceae, are highly metabolically versatile [21–25]. Based on metagenome-assembled genomes (MAGs), many of these taxa are capable of simultaneously or alternately using multiple energy sources (e.g., organic carbon, sulfide, hydrogen, sunlight), carbon sources (organic carbon, carbon dioxide), and metabolic strategies (e.g., aerobic respiration, denitrification, fumarate reduction, fermentation) [21–23]. Continual variations in oxygen levels in these sediments select for facultative anaerobes; in situ evidence suggests that some bacteria can even perform aerobic and anaerobic respiration simultaneously, for example aerobic denitrifiers [26, 27]. In contrast, obligate anaerobes such as sulfate reducers and methanogens are thought to be inhibited by transient oxygenation, despite their preferred electron donors and acceptors being available [21, 23].

The physical features and microbial communities of permeable sediments in turn influence biogeochemical processes. Permeable sediments exposed to high tidal disturbance are minimally stratified in geochemistry and thus carbon mineralization does not follow the classical ‘redox cascade’ established for cohesive sediments [3]. In sediments from Port Philip Bay, Australia, fermentation is the dominant pathway of carbon mineralization under anoxic conditions and is largely uncoupled from anaerobic respiration [23, 28]. This reflects that facultative fermenters are the dominant community members and switch to hydrogenogenic fermentation when preferred electron acceptors such as oxygen and nitrate are limiting. In turn, fermentation products accumulate in situ and ex situ due to low levels of sulfate reducers and other obligate anaerobes [23]. Likewise, multiple studies have inferred that rates of denitrification exceed those of dissimilatory nitrate reduction to ammonium (DNRA), again suggesting the predominance of processes associated with facultative anaerobes rather than obligate anaerobes [29–31]. Nevertheless, there is evidence of some variation in the anaerobic respiratory processes between sediments, with high levels of sulfate reducers reported in some environments [13, 32, 33]. For example, Probandt et al. observed that the abundance of sulfate-reducing Desulfobulbaceae and Desulfobacteraceae in surface sands decreases with permeability [18]. Thus, differences in mixing levels between different sediments, for example due to variations in hydrodynamic forcing or sediment topography, likely influence microbial community assembly and in turn biogeochemical processes.

In this work, we build on this conceptual framework to investigate how the microbiology and biogeochemistry of permeable sediments varies across a disturbance gradient. To do so, we sampled sediment cores from three sites along a 2.1 km stretch of beach in Port Philip Bay, Australia, which differed in levels of hydrodynamic forcing: one site was fully exposed to wave disturbance (site A), whereas the others were either moderately (site B) or highly (site C) buffered by a breakwater (Fig. S1). Based on the above framework, we developed three key testable hypotheses for how these sites may differ: (1) Less disturbed sites will be more stratified in geochemistry and microbial community structure; to test this, we combined geochemical analysis with 16S rRNA gene-based community profiling of sediment core subsections. (2) More microbial specialists, including obligate anaerobes, will be present in less disturbed sites; to test this, we combined deep metagenomic sequencing of each site, yielding 169 MAGs, with microbial community analysis and biogeochemical assays. (3) Carbon mineralization processes will be more tightly coupled to anaerobic respiration in less disturbed sites; we tested this by performing microcosm experiments to measure rates of hydrogenogenic fermentation, sulfate reduction, denitrification, and DNRA across the sediments. Our investigations suggest that these predictions are partially correct, though some unexplained patterns were observed. Community composition and metabolic genes showed strong correlations with the disturbance level of a given sample within a depth profile (as inferred by distance to sulfidic layer), as well as by site or depth alone. These findings also enhance knowledge of the processes and microorganisms controlling marine biogeochemical cycling.

## Materials and methods

### Sediment sampling

Permeable sediments were sampled across a 2.1 km stretch of Port Phillip Bay, Australia. Three different sampling sites, site A (Middle Park Beach; 37.851342°S, 144.954377°E), site B (Cummings Reserve; 37.856283°S, 144.964258°E), and site C (St. Kilda Pier; 37.863159°S, 144.971026°E), were selected based on their different levels of hydrodynamic exposure due to the St. Kilda Breakwater providing shelter from the prevailing westerly and southerly wind directions (Fig. S1). The three sites were sampled on eight different dates for different purposes: preliminary community and geochemical profiling (28/02/2018); complete community and geochemical profiling (14/06/2018); measurement of mixing layer depth and grain size distribution (14/06/2018, 26/02/2019, 11/03/2019); and microcosm experiments to analyze carbon fixation (07/08/2018), H_2_ metabolism (20/06/2018), sulfide production (26/02/2019), denitrification (16/03/2020), DNRA (16/03/2020), and nitrification (21/03/2021). We confirmed that the sediments varied in hydrodynamic forcing by measuring mixing layer depth and grain size distribution at three dates (Table S1). To measure mixing layer depth, cores of 30 cm were collected and photographed, and the depth of the darker sulfidic layer was quantified using ImageJ [34]. To determine grain size distribution, 100 g sediments (dry weight) were collected at each of the three sampling dates. Sand was progressively separated using eight sieves of different sizes (4, 2, 1, 0.85, 0.5, 0.25, 0.125, and 0.063 mm). Median grain size (D_50_) was calculated based on the equation of Ferguson & Church [35, 36]. Five individual sediment cores of 30 cm depth were collected from each site at two dates. Cores were kept on ice until delivery to the laboratory and then immediately sliced every 2 cm, with the eight sections from the top 0 to 16 cm used for downstream analysis. For each site, two cores were used for DNA extraction and chlorophyll *a* measurement, and three other cores were used for sulfide and ammonium measurements.

### Geochemical measurements

Sulfide and ammonium content were measured immediately after sediment sectioning. Approximately 30 g from each sediment slice was transferred into 30 ml of N_2_-purged artificial seawater. Following stirring for 10 s, the supernatant was extracted using a syringe for further analysis. Free sulfide concentrations were quantified using the methylene blue method with a GBC UV-Visible 918 Spectrophotometer at 670 nm as previously described [37]. For acid-volatile sulfide (AVS) measurements, sediments were stored frozen and then analyzed as previously described [38]. Briefly, after samples were thawed and homogenized, 0.1 g of sediment was treated with an acidified methylene blue reagent, centrifuged, and stored in the dark for 90 min before analysis. This process results in the conversion of AVS to free sulfide, which is then quantified in the same way as free sulfide. Ammonium concentrations were determined by the phenate method using a Lachat Quickchem 8000 Flow Injection Analyzer at 630 nm as previously described [39]. Chlorophyll *a* was extracted and quantified as previously described using a Hitachi U-2800 spectrophotometer (Hitachi High-Technologies Corporation, Tokyo, Japan) [21, 40].

### DNA extraction and microbial community analysis

DNA was extracted from 0.3 g of each 2 cm sediment slice using the MoBio PowerSoil Isolation kit according to the manufacturer’s instructions (https://www.qiagen.com/au/resources/download.aspx?id=5c00f8e4-c9f5-4544-94fa-653a5b2a6373&lang=en). In total, 48 samples were sequenced and analyzed (3 sites × 2 cores × 8 depths). Samples were eluted in DNase- and RNase-free UltraPure Water (Thermo Fisher Scientific). A sample-free negative control, containing only UltraPure Water, was also extracted. Nucleic acid purity and yield were confirmed using a Nanodrop 1000 spectrophotometer and a Qubit 2 fluorometer. To estimate the number of bacteria and archaea present in each sample, quantitative PCR (qPCR) of the 16S rRNA gene was performed using universal primer pairs F515 and R806 [41]; assays were performed using a 96-well plate in a pre-heated LightCycler 480 Instrument II (Roche, Basel, Switzerland) and 16S rRNA gene copy number was quantified against a serially diluted pMA plasmid containing the *Escherichia coli* 16S rRNA gene as previously described [21]. For amplicon sequencing, the V4 hypervariable region for 16S rRNA gene was amplified using the primer pairs F515 and R806 [41]. Amplicons were subject to 2 × 300 bp sequencing on a MiSeq platform (Illumina) at the Australian Centre for Ecogenomics (ACE), the University of Queensland. Amplicon sequences were then processed using the pipeline provided by ACE (https://wiki.ecogenomic.org/doku.php?id=amplicon_pipeline_readme). For sequencing runs did not attain requested depth, the same library was re-sequenced and combined in QIIME2. The forward reads were trimmed to 250 base pairs and low quality reads were removed using Trimmomatic [42]. All reads were then subjected to de-noising using the DADA2 pipeline [43] in QIIME2 [44]. A total of 2,969,857 reads from 48 samples were obtained from the dataset (Table S2), with reads removed by the DADA2 pipeline provided in Table S3. The negative control did not yield quantifiable DNA or detectable amplicons on agarose gels, suggesting minimal contamination during sample processing, and was not sequenced. For taxonomic assignment, all reference reads that matched the F515/R806 primer pair were extracted from the Genome Taxonomy Database (GTDB) release 04-RS89 [45] and used to train a naïve Bayes classifier by using the fit-classifier-naive-Bayes function with default parameters.

### Biodiversity analysis

All statistical analysis and visualizations were performed with R software version 3.6.2 (December 2019) using the packages phyloseq ggplot2 [46] and microbiome [47]. The outputs from QIIME2, without rarefaction, were used to analyze community composition at phylum, order, family, and genus levels. The relative abundance of each assigned order, family, and genus was compared by site (categories: site A, site B, site C) and depth (categories: 0–4 cm (shallow), 4–10 cm (medium), 10–16 cm (deep)) using one-way ANOVAs. In addition, linear regressions were performed to test how the relative abundance of each assigned order, family, and genus varied relative to the disturbance level of each individual sample (as inferred by their distance in cm relative to the average depth of the dark sulfidic layer). To analyze alpha diversity and beta diversity, all sequences were rarefied at 10,000 sequences per sample using the phyloseq function *rarefy_even_depth()* to account for the difference in library sizes [48]. The rarefied dataset retained 907,977 reads (30%) across 43 of the 48 original samples (90%). Alpha diversity was calculated using several metrics, including Shannon index and Simpson index. Significant differences in Shannon index between sites and depths were tested using an ANOVA (one-way analysis of variance) with Tukey’s post hoc tests. Beta diversity was calculated using weighted UniFrac distances [49] of log-transformed data and visualized using a principal coordinate analysis (PCoA) plot. A pairwise analysis of similarities (ANOSIM) was also used to test for significant differences in community composition. First, a nested permutational multivariate analysis of variance (PERMANOVA) was performed using 999 permutations to test for significant differences. Second, a beta dispersion test (PERMDISP) was used to ascertain if observed differences were influenced by dispersion. An analysis of composition of microbiomes (ANCOM) [50] was also performed in QIIME2 to detect differential abundance of genera between sites A and C. This analysis compares the centered log-ratio (clr) transformed data of a specific taxon to the rest of taxa between two distinct environments.

### Shotgun metagenomic assembly and binning

DNA samples extracted from shallow (0–2 cm), intermediate (6–8 cm), and deep (14–16 cm) sediment slices from each of the three sites were subject to shotgun metagenomic sequencing. Metagenomic shotgun libraries were prepared for each sample using the Nextera XT DNA Sample Preparation Kit (Illumina Inc., San Diego, CA, USA) and sequencing was performed on an NextSeq500 platform (Illumina) with a 2 × 150 bp High Output run. Sequencing yielded 523,268,618 read pairs across the nine metagenomes. The BBDuk function of the BBTools v38.51 (https://sourceforge.net/projects/bbmap/) was used to clip contaminating adapters (k-mer size of 23 and hamming distance of 1), filter PhiX sequences (k-mer size of 31 and hamming distance of 1), and trim bases with a Phred score below 20 from the raw metagenomes. After removing resultant reads with lengths shorter than 50 bp, 450,359,308 high-quality read pairs were retained for downstream analysis. Reads were assembled individually with MEGAHIT v1.2.9 [51] (-k-min 27, -k-max 127, -k-step 10, -min-contig-len 500). To improve recovery of metagenomic bins, metagenomes from samples of high hydrodynamic disturbance (A0-2, A6-8, B0-2), intermediate hydrodynamic disturbance (A14-16, B6-8, C0-2), and low hydrodynamic disturbance (B14-16, C6-8, C14-16) were also assembled collectively using MEGAHIT with same parameters as above. Bowtie2 v2.3.5 [52] was used to map short reads back to assembled contigs using default parameters to generate coverage profiles. Subsequently, genomic binning was performed using Autometa (09/2019) [53], CONCOCT v1.1.0 [54], MaxBin2 v2.2.6 [55], and MetaBAT2 v2.15 [56] on contigs with lengths over 2000 bp. Resulting bins from the same assembly were then dereplicated using DAS_Tool v1.1.2 [57]. RefineM v0.0.25 was used to remove potentially contaminating contigs with incongruent genomic and taxonomic properties in the bins [58]. Applying a threshold average nucleotide identity of 99%, bins from different assemblies of each hydrodynamic disturbance category were consolidated to a non-redundant set of MAGs using dRep v2.5 [59]. Completeness and contamination of MAGs were assessed using CheckM v1.1.2 [60]. In total, 30 high quality (completeness > 90% and contamination < 5%) and 139 medium quality (completeness > 50% and contamination < 10%) [61] MAGs were recovered. Their corresponding taxonomy was assigned based on GTDB release 05-RS95 by GTDB-Tk v1.0.2 [45]. Open reading frames (ORFs) in MAGs were predicted using Prodigal v2.6.3 [62].

### Shotgun metagenome community and functional analysis

Bacterial, archaeal, and eukaryotic community composition of the metagenomes was profiled using phyloFlash v.3.4 [63]. All quality-filtered reads were screened for the small subunit ribosomal RNA gene (SSU rRNA) sequences and assembled with the command *phyloFlash.pl* and the option –almosteverything. The SSU Ref NR99 database from the SILVA release 138 served as the reference for the sequence searching and taxonomy assignment of SSU reads to Nearest Taxonomic Units (NTUs) [64]. To estimate the metabolic capability of the sediment communities, metagenomes and derived genomes were searched against custom protein databases (10.26180/c.5230745) of representative metabolic marker genes [65] using DIAMOND v.0.9.31 (query cover > 80%) [66]. Searches were carried out using all quality-filtered unassembled reads with lengths over 140 bp and the ORFs of the 169 MAGs. These genes are involved in respiration (AtpA, NuoF, SdhA, CoxA, CcoN, CyoA, CydA), sulfur cycling (AsrA, FCC, Sqr, DsrA, Sor, SoxB), nitrogen cycling (AmoA, HzsA, NifH, NarG, NapA, NirS, NirK, NrfA, NosZ, NxrA, NorB), iron cycling (Cyc2, MtrB, OmcB), reductive dehalogenation (RdhA), photophosphorylation (PsaA, PsbA, energy-converting microbial rhodopsin), methane cycling (McrA, MmoA, PmoA), hydrogen cycling (large subunit of NiFe-, FeFe-, and Fe-hydrogenases), formate oxidation (FdhA), carbon monoxide oxidation (CoxL, CooS), fumarate reduction (FrdA), arsenic cycling (ARO, ArsC), selenium cycling (YgfK), and carbon fixation (RbcL, AcsB, AclB, Mcr, HbsT, HbsC) [67–69]. Results were further filtered based on an identity threshold of either 80% (PsaA), 75% (HbsT), 70% (AtpA, PsbA, ARO, YgfK), 60% (NuoF, RbcL, CoxL, AmoA, NxrA, MmoA, FeFe-hydrogenase, group 4 NiFe-hydrogenase), or 50% (all other databases). Subgroup classification of reads was based on the closest match to the sequences in databases. MtrB in MAGs was screened additionally using hidden Markov models (HMM) [70], with search cutoff scores as described previously [71]. Read counts to each gene were normalized to reads per kilobase million (RPKM) by dividing the actual read count by the total number of reads (in millions) and then dividing by the gene length (in kilobases). In order to estimate the gene abundance in the microbial community, high-quality unassembled reads were also screened for the 14 universal single copy ribosomal marker genes used in SingleM v.0.12.1 and PhyloSift [72] by DIAMOND (query cover > 80%, bitscore > 40) and normalized as above. Subsequently, the average gene copy number of each gene in the community was inferred by dividing the read count for the gene (in RPKM) by the mean of the read count of the 14 universal single copy ribosomal marker genes (in RPKM). Linear regressions were performed to test how the relative abundance of each gene varied relative to the disturbance level of each individual sample (as inferred by their distance in cm relative to the average depth of the dark sulfidic layer).

### Sulfate reduction assays

Anoxic slurry experiments were performed to compare the rates of H_2_ metabolism and sulfate reduction between the three sites. Sediments of 0–10 cm depth were collected from site A, B, and C for H_2_ measurements and sulfide measurements. Each slurry comprised a 160 mL serum vial containing 30 g of sieved sand (wet weight) and 70 mL of seawater (filtered on 0.45 µm Whatman membrane filters). The serum vials were sealed with butyl rubber stoppers and Wheaton closed-top seals. An autoclaved vial was used as the control group. All vials were purged with high-purity helium gas and covered with aluminium foil to create dark anoxic conditions. The headspace of the vial was amended with 100 ppmv H_2_ and, for the glucose treatment, 1 mM glucose. All vials were incubated on a shaker (100 rpm) at room temperature. For H_2_ measurements, a 2 mL subsample was collected from headspace every 24 h and analyzed using a VICI Trace Gas Analyzer Model 6K (Valco Instruments Co. Inc., USA) fitted with a pulsed discharge helium ionization detector that was configured and calibrated as previously described [73]. Three independent slurries were performed per treatment. Headspace H_2_ mixing ratios were converted to dissolved H_2_ concentrations in the slurries by applying Henry’s law. After two weeks of incubation, DNA was extracted from the sediments and subject to 16S rRNA gene amplicon sequencing as described above. For free sulfide measurements, a total of 8 mL of seawater was extracted from each slurry and filtered for spectrophotometric analysis using the methylene blue method [37]. AVS measurements were performed as described above.

### Carbon fixation assays

Shallow (0–10 cm) and deep (10–20 cm) sediments were collected from sites A, B, and C for comparison of rates of dark carbon fixation. 30 g sediment (wet weight) and 70 mL seawater in 160 mL serum vials were sealed (ambient air headspace) with butyl rubber stoppers and Wheaton closed-top seals. Slurries either remained unamended (native electron donors or were supplemented with electron donors 200 µM sodium sulfide (Na_2_S·9H_2_O) or 200 µM ammonium chloride (NH_4_Cl). Vials containing autoclaved sediments were used as controls. Radiolabelled sodium bicarbonate solution (NaH^14^CO_3_, Perkin Elmer, 53.1 mCi nmol^−1^) was administered to a concentration of 300 µM to each slurry. Slurries were incubated for 18 h in a light proof box (175 rpm, room temperature). After this time, the slurry was adjusted to pH 2 with 5 mL 1 M HCl to stop carbon fixation and acidify unfixed bicarbonate, before centrifugation at 1000 × *g* for 10 min. Overlying seawater was discarded before sediment was left to dry in oven (80 °C) and then the acidification was repeated. Sediment was weighed into scintillation vials, combined with scintillation cocktail (EcoLume), and radioisotope analysis conducted using a liquid scintillation spectrometer (Tri-Carb 2810 TR, Perkin Elmer). The scintillation counts from autoclaved controls were subtracted from all samples. Initial seawater (preserved with 6 % w/v HgCl_2_) was analyzed for dissolved inorganic carbon (DIC analyzer, Apollo SciTech) and used in combination with the specific radioactivity of the bicarbonate solution to calculate the amount of ^14^C fixed per vial.

### Denitrification, DNRA, and nitrification assays

Shallow (0–5 cm) and deep (20–25 cm) sediment samples were collected from sites A, B, and C. Slurries containing 30 g sediment and 100 mL filtered seawater were prepared in 160 mL serum vials, which were then crimp-sealed with a butyl rubber septum. For denitrification and DNRA assays, each slurry was amended with Na^15^NO_3_ (>98% ^15^N) to a final concentration of 1 mM and purged with argon to create anoxic conditions. The slurries were continuously mixed on a shaker table at 125 rpm for the duration of the incubation period. To determine denitrification rates, at each time point, 3 mL headspace samples (containing ^15^N-N_2_) were removed and replaced with 3 mL argon. ^15^N-N_2_ was analyzed using a Sercon 20–22 continuous flow isotope ratio mass spectrometer coupled to a gas chromatograph as previously described [74]. To determine DNRA rates and for geochemical analyses, 15 mL filtered seawater (containing ^15^N-NH_4_^+^) was removed and replaced with 15 mL Ar-purged filtered seawater amended with 1 mM Na^15^NO_3_. For iron analysis, 2 mL filtered samples were added to 0.5 mL of 10 mM ferrozine and analyzed as described [75]. For sulfide analysis, 2 mL samples were preserved with 10% v/v 28 mM Zn acetate and analyzed as described above. 7.5 mL samples for ^15^N-NH_4_^+^ analysis were transferred to a 12.5 mL gas tight exetainer and preserved with 250 μL ZnCl_2_. The samples were purged with He to remove background N_2_ before 200 μL alkaline hypobromite was added to each sample to convert ^15^N-NH_4_^+^ to ^15^N-N_2_ as described [76]. To ensure quantitative conversion of NH_4_^+^ to N_2_, samples were shaken at 130 rpm for 16 h prior to instrumental analysis using the gas chromatograph-isotope ratio mass spectrometer. Denitrification and DNRA from the slurry experiments were estimated based on the accumulation of ^15^N-N_2_ and ^15^NH_4_^+^, respectively, over eight days after ^15^N-NO_3_^−^ addition. To measure nitrification, oxic slurries were amended with 50 µM NH_4_Cl. NO_2_^−^ and NO_3_^−^ concentrations in the nitrification assay were determined by the Griess method [77] using a Lachat Quickchem 8000 Flow Injection Analyzer. Rates of nitrification were calculated from linear regression of NO_2_^−^ and NO_3_^−^ increase over time. The significance of differences in denitrification, DNRA, and nitrification were tested by ANCOVA using R (v 4.0.3).

## Results

### Geochemical stratification and microbial abundance are higher in less disturbed permeable sediments

**Fig. 1:**
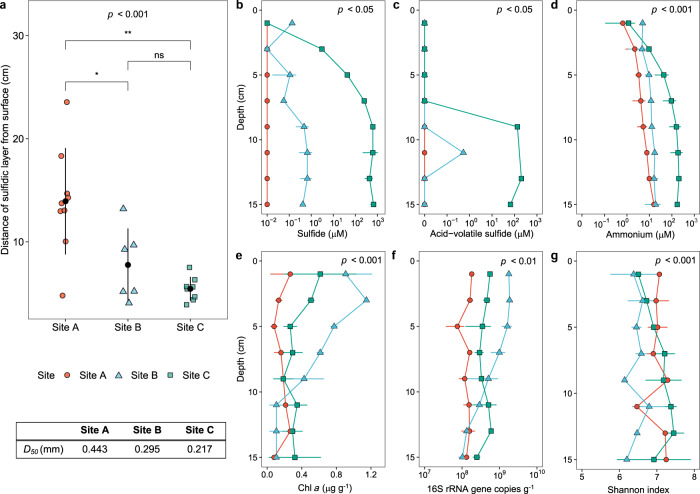
Differences in geochemical stratification and microbial abundance between sites. **a** Depth of the sediment mixing layer, based on sampling across three dates. The average median grain size (D_50_; in mm) is tabulated, with full details in Table S1. Concentrations of (**b**) free sulfide, (**c**) acid-volatile sulfide, and (**d**) ammonium are shown relative to sediment depth for each of the three sites. Also shown are (**e**) chlorophyll *a* content, (**f**) 16S rRNA gene copy number, and (**g**) alpha diversity based on Shannon index relative to sediment depth for each of three sites. Dot points show averages and error bars show standard deviations from either three (**b**, **d**, **e**) or two (**f**, **g**) sediment cores. For AVS measurements, one replicate was performed per slurry and hence error bars are not shown. One-way ANOVAs were used to test significant differences in parameters between sites.

We confirmed that the three sediment sites (Fig. S1) differed in levels of hydrodynamic disturbance by measuring mixing layer depth and grain size distribution at three different sampling dates. The depth and variability of the mixing zone were greatest for the highly disturbed site as expected (Fig. 1a); the average depth of the mixing zone (i.e., depth to the black sulfidic layer) shifted from 13.9 ± 5.2 cm for site A compared to 7.8 ± 3.5 cm for site B and 5.4 ± 1.1 cm for site C (*p* < 0.001, one-way ANOVA). This supports previous inferences that greater hydrodynamically-driven porewater flow results in stronger sediment mixing and deeper oxygen penetration overall, though in a spatiotemporally heterogeneous manner [12, 13]. In addition, grain size distribution varied as anticipated. All three sediments were mainly comprised of sand and gravel grains, with median grain size larger in site A (D_50_ = 443 µm) than sites B (295 µm) and C (217 µm) (Table S1). Altogether, these findings suggest that the highly exposed site A is the most permeable, disturbed, and aerated of the sediments, whereas the breakwater-protected site C is far less so and site B has intermediate characteristics.

Consistent with our hypotheses, geochemical stratification was more pronounced for the less disturbed sites. Reflecting this, significant differences in the concentrations of sulfide (*p* < 0.05, one-way ANOVA) and ammonium (*p* < 0.001, one-way ANOVA) were detected between the sites. Free sulfide increased with depth to high concentrations in site C (av. 321 μM) and moderate concentrations in site B (av. 309 nM) (Fig. 1b), indicating activity of dissimilatory sulfate reducers. In contrast, sulfide was below detection limits at all sampled depths for site A (Fig. 1b), in agreement with previous observations that hydrogenotrophic sulfate reduction is inhibited and aerobic sulfide oxidation occurs at rapid rates in highly disturbed sediments [21, 23, 28]. Acid-volatile sulfide (AVS) measurements followed similar patterns (Fig. 1c). In addition, ammonium accumulated in site C but to a lesser extent at the other sites (Fig. 1d), suggesting ammonia production from organic matter mineralization and DNRA predominates over nitrification in more anoxic sediments. Linear regression analysis confirmed concentrations of free sulfide (*p* < 0.0001), AVS (*p* = 0.038), and ammonium (*p* < 0.0001) were significantly negatively correlated with disturbance level, as inferred by average distance of each sample (in cm) to the sulfidic layer (Fig. S2).

Chlorophyll *a* content, which indicates cyanobacterial and eukaryotic photosynthesis, decreased with depth as expected given variations in light exposure (Fig. 1e). Somewhat surprisingly, site A contained twofold lower chlorophyll *a* content than the other sites across the depth profile (*p* < 0.001, one-way ANOVA), suggesting regular temporal transitions from light oxic to dark anoxic conditions in these sediments exclude photoautotrophs. However, trends in the abundance and diversity of bacterial and archaeal communities were complex and not clearly related to differences in disturbance level. Microbial abundance (inferred from 16S rRNA gene copy number; Table S1) was relatively high across all sites (av. 4.1 × 10^8^ copies per gram of sediment), suggesting all sediment subsections harbor abundant communities well-adapted to their respective environmental conditions and disturbance regimes. For unclear reasons, abundance was the highest and most variable at site B, and the lowest and least variable at site A (Fig. 1f). In contrast, bacterial and archaeal diversity of each sample (Shannon index and estimated richness based on 16S rRNA gene amplicon sequencing; Tables S2 & S4) was highest for site C, lowest for site B, and considerably varied within the depth profiles (Fig. 1g). Likewise, fewer amplicon sequence variants (ASVs) were observed across the entire sediment profiles for site B (13651, 7515, and 18288 ASVs detected at sites A, B, and C respectively). No correlations were observed between sample disturbance level with microbial abundance (*p* = 0.31) or Shannon index (*p* = 0.36) based on linear correlation analysis (Fig. S2). One explanation for these observations is that the intermediate level of disturbance in site B increases carrying capacity by enabling extensive aerobic and anaerobic growth of different community members on the various electron donors available, in contrast to the relatively oxic site A and anoxic site C, but in turn reduces diversity by intensifying competition.

### Community composition is highly differentiated by sediment disturbance level

**Fig. 2:**
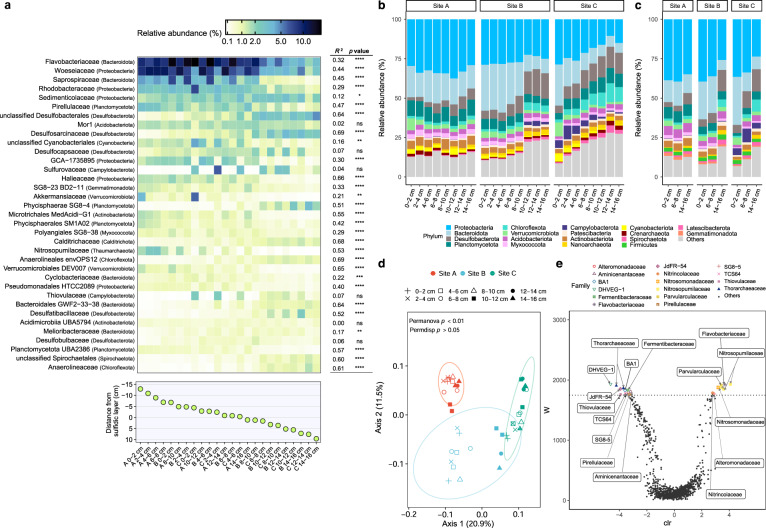
Differences in bacterial and archaeal community composition between sites. **a** Comparison of the relative abundance of the 35 most abundant bacterial and archaeal families, based on 16S rRNA gene amplicon sequencing, of sediment cores sectioned into 2 cm slices. Samples are arranged by disturbance level, based on their average distance (in cm) from the sulfidic layer. *p* values denote whether a linear regression line of family relative abundance versus sample disturbance sample is significantly non-zero. * *p* < 0.05, ** *p* < 0.01, *** *p* < 0.001, **** *p* < 0.0001. **b** Phylum-level bacterial and archaeal composition, based on 16S rRNA gene amplicon sequencing, of sediment cores sectioned into 2 cm slices. Results are averaged based on two independent sediment cores. **c** Phylum-level bacterial and archaeal composition, based on 16S rRNA gene reads in shotgun metagenomes, of a representative subset of samples from the sediment cores. **d** Beta diversity of communities based on weighted Unifrac analysis of 16S rRNA gene amplicon sequencing data. Samples are visualized by principal coordinates analysis (PCoA) with colors used to denote site and shapes used to denote sediment depth. The ellipse represents 95% confidence intervals. **e** Differential abundance of rare and common genera between sites A and C based on analysis of composition of microbiomes (ANCOM). In the volcano plot, the *F*-score represents the log-fold change of the centered log ratio (clr) transformation, with positive values representing taxa more abundant at site A and negative values indicating taxa more abundant in C. The W-statistic determines whether differential abundance is significant. The false discovery rate (FDR) of 0.05 was controlled by Benjamini–Hochberg correction.

Bacterial and archaeal community composition was analyzed in duplicate sediment cores from each site by 16S rRNA gene amplicon sequencing (Fig. 2). Across the 48 samples, 30,830 ASVs were detected from 82 cultured or candidate phyla (Table S2). Beta diversity analysis (weighted UniFrac) confirmed community composition differed between sites (*p* < 0.01, PERMANOVA) (Fig. 2c). Based on a PCoA visualization (Fig. 1d), samples most strongly clustered by disturbance level along axis 1 (explaining 20.9% of the variation): community composition was similar between the most disturbed layers of site B (0–4 cm) with those of site A, and between the least disturbed layers of site B (10–16 cm) with those of site C. For site A, communities exhibited weak minimal depth stratification (Fig. 2b, 2d), consistent with the observation hydrodynamic mixing selects for habitat generalists [21]. However, contrary to our original hypothesis, depth stratification was greatest for the moderately disturbed site B (Fig. 2b, d); this reflects that large community shifts occur between mixing and sulfidic zones, but there are many shared taxa within each of these zones. Overall, these findings suggest differences in hydrodynamic forcing between samples, due to the combination of site and depth, controls microbial community assembly.

Disturbance level also strongly correlated with variations in the relative abundance of most phyla, orders, families, and genera between the sites (Fig. 2; Table S5). The most abundant families were Flavobacteriaceae (Bacteroidota; av. 8.9 ± 4.8% relative abundance) and Woeseiaceae (Proteobacteria; av. 6.9 ± 3.5%), consistent with reports that they are metabolically flexible habitat generalists [19, 21–23, 25]; their abundance was higher than all other families in all samples except six from the deeper depths of site C (Fig. 2a, b; Table S5). Several other families were also abundant and prevalent, especially in more disturbed sites, including within phyla Bacteroidota (Saprospiraceae, Cyclobacteriaceae), Proteobacteria (Rhodobacteraceae, Sedimenticolaceae, GCA-1735895, Helieaceae), and Planctomycetota (Pirellulaceae, Phycisphaerae families SG8-4 and SM1A02) (Fig. 2a, b; Table S5). Conversely, lineages reported to be obligate anaerobes were enriched in the least disturbed sites and deepest sediment depths. These include both named and novel families within the sulfate-reducing class Desulfobacteria [78], as well as putative fermenters within Bacteroidia, Anaerolineae, and Spirochaetia [79, 80] (Fig. 2a, b; Table S5). Although we observed considerable differentiation in the relative abundance of families, most families were still present across multiple sites (average occupancy of 19 out of 48 samples) and 50 of them were shared across all sites, including all the previously named families (Table S5). While occupancy was lower at the ASV level (average occupancy of 3.9 out of 48 samples), 15 abundant ASVs were shared across all samples (including several ASVs each within the families Flavobacteriaceae, Woeseiaceae, and Desulfocapsaceae) (Table S2). Overall, these findings support our hypothesis that decreased disturbance select for more obligately anaerobic taxa more typical of cohesive sediments, though suggest that stratification is relatively modest given many bacteria can adapt to a wide range of disturbance levels.

We performed statistical tests to analyze variations in the relative abundance of the 35 most abundant named families (Fig. 2a). 31 families significantly varied in relative abundance between sites, and 20 significantly varied between depths (*p* < 0.05, one-way ANOVA) (Fig. S3; Table S5). Reflecting that disturbance level is a nested variable, we also used linear regressions to determine whether the relative abundance of each family varies continuously with disturbance level, as inferred from the distance of each sediment sample from the sulfidic layer (Fig. 2a; Fig. S4). 18 families significantly increased (e.g., Flavobacteriaceae, Woeseiaceae) and 11 families significantly decreased (e.g., Desulfosarcinaceae, Anaerolineaceae) with disturbance level (*p* < 0.0001 for 24 families). However, it should be noted that the coefficients of determination (*R*^2^) considerably varied between these families (Fig. S4; Table S5), suggesting disturbance level interacts with other factors to control their relative abundance. Two families (Sulfurovaceae, Thiovulaceae) were only abundant at the oxic-sulfidic interface of site C (Fig. S3), in line with their reported for aerobic chemolithoautotrophic growth on high levels of sulfide such as those present at this site (Fig. 1b) [81]. Two other families (Acidobacteriota Mor1, Acidimicrobiia UBA5794) exhibited no clear patterns with disturbance level (Fig. 2a). We additionally performed an analysis of composition of microbiomes (ANCOM) [50] to detect significant differences in the relative abundance of different genera, including members of the rare biosphere (Fig. 2d). Interestingly, archaea were among the strongly differentiated: putative aerobic ammonium-oxidizing Nitrosopumilaceae [82, 83] were most enriched in the more disturbed sites, whereas members of the putatively anaerobic orders Thorarchaeaceae [84], Bathyarchaeia BA1 [85], and Thermoplasmatota DHVEG-1 were enriched in the anoxic sites (Fig. 2d; Fig. S3). Methanogens (e.g., Methanosarcinaceae) were also enriched in less disturbed sediments (relative abundance of 0.56% at site C, 0.83% at 10–16 cm depth; Table S5). Consistent with these observations, the average occupancy of archaeal taxa (15.8 at family level, 2.6 at ASV level) was lower than for bacteria (19.0 at family level, 4.0 at ASV level) (Tables S2 & S5).

Finally, we profiled composition of the entire microbial community by sequencing metagenomes of each site at three depths (0–2 cm, 6–8 cm, 14–16 cm; Table S6) and taxonomically assigning ribosomal small subunit genes using phyloFlash (Tables S7 & S8). Bacterial and archaeal community composition was similar based on metagenomic and amplicon sequencing (Fig. 2b, c; Table S8). Diverse eukaryotes were also detected in the metagenomes, including putatively photoautotrophic diatoms, dinoflagellates, charophytes, and euglenids; in support of the chlorophyll *a* data (Fig. 1e; Table S1), their abundance relative to bacteria and archaea decreased with sediment depth (Fig. S5; Table S7).

### Capacity for aerobic respiration, anaerobic respiration, and fermentation varies with disturbance level

**Fig. 3:**
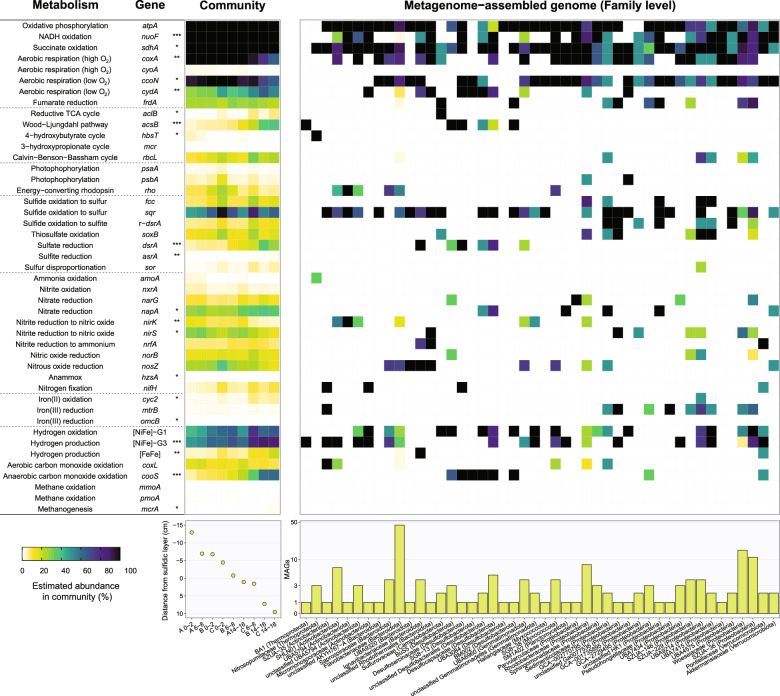
Metabolic capacity of microbial communities. Homology-based searches were used to detect key metabolic genes in nine metagenomes and 169 derived metagenome-assembled genomes. The left heatmap shows the percentage of community members in each metagenome predicted to encode each gene based on the short reads. Hits were normalized to gene length and single-copy ribosomal marker genes. Samples are arranged by their disturbance level (bottom left panel), as inferred from their average distance (in cm) to the sulfidic layer (more disturbed sites have more negative distances and less disturbed sites have more positive distances relative to sulfidic layer). *p* values denote whether a linear regression line of gene community abundance versus sample disturbance sample is significantly non-zero. * *p* < 0.05, ** *p* < 0.01, *** *p* < 0.001. The right-hand heatmap show the proportion of metagenome-assembled genomes from each family that are predicted to encode each metabolic marker gene. The histogram (bottom right panel) shows the number of MAGs per family on a logarithmic scale.

We analyzed the nine metagenomes to gain mechanistic insights into observed differences in community structure and geochemical parameters (Tables S6 & S9). The distribution of 50 metabolic marker genes involved in energy acquisition, electron acceptor utilization, and carbon fixation was determined across the metagenomic short reads (Table S9). In line with previous studies [21, 23], the high abundance of various marker genes (Fig. 3) suggests bacteria within the sediments can switch between aerobic respiration (using terminal oxidases), anaerobic respiration (via denitrification steps), and fermentation (using evolving hydrogenases). There is also wide capacity for organic carbon, sulfide, hydrogen, carbon monoxide, and formate oxidation (Fig. 3). The relative abundance of several genes significantly varied with disturbance level (*p* < 0.05, linear regression), as inferred from the distance of each sediment sample from the sulfidic layer (Table S9). These include various genes associated with anaerobic metabolism that decreased with disturbance level. Notably, there was a significant increase of at least fivefold in the marker genes for sulfate reduction (*dsrA*, *asrA*), fermentation (FeFe-hydrogenases), the Wood-Ljungdahl pathway (*acsB*, *cooS*), and methanogenesis (*mcrA*) from the most to least disturbed samples (*p* < 0.05, linear regression). In contrast, marker genes associated with aerobic growth (*coxA*), archaeal nitrification (*hbsT*), and surprisingly stepwise nitrite reduction (*nirK*, *nirS*) increased in abundance by at least twofold from the least to most disturbed samples (*p* < 0.05, linear regression) (Fig. 3; Fig. S6). Alongside the community analysis (Fig. 2), these findings support our second hypothesis that metabolically flexible habitat generalists are abundant throughout the sediments, though metabolically constrained anaerobic specialists are relatively enriched in less disturbed sediments.

In order to link community members to metabolic processes, the metagenomes were individually assembled and co-assembled based on sediment disturbance level. Binning yielded 169 high- or medium-quality metagenome-assembly genomes (MAGs) [61] from ten phyla and 46 families (Fig. 3; Table S10), including 18 of the 35 most abundant families (Fig. 2). Given the MAGs vary in completeness, we inferred the potential lifestyles of the families present based on what metabolic genes were present rather than absent, in conjunction with referencing previous literature regarding each lineage. Abundant habitat generalists, such as Flavobacteriaceae, Woeseiaceae, Rhodobacteraceae, and Saprospiraceae, encode diverse metabolic genes; in line with previous findings [21, 23], they’re variably capable of shifting between aerobic organotrophic respiration, and sulfide-dependent autotrophic growth under light oxic conditions to performing hydrogenogenic fermentation and denitrification steps when sediments become dark and anoxic (Fig. 3; Table S10). The MAGs also support the divergent distributions of two archaeal families highlighted by the ANCOM analysis (Fig. 2e): whereas the Nitrosopumilaceae MAGs are likely to be aerobic specialists that oxidize ammonia, Bathyarchaeia BA1 are predicted to be anaerobic acetogens, in support of previous reports [21, 82, 86] (Fig. 3; Table S10).

The MAG-level analysis rationalizes the strong differentiation in sulfur metabolism between samples. In support of recent findings [21, 23, 32, 87], the MAGs suggest multiple families from the phylum Desulfobacterota are the dominant sulfate reducers in permeable sediments (Fig. 3b). Genes encoding enzymes mediating sulfite reduction (dissimilatory sulfite reductase; *dsrA*) were detected in seven MAGs, usually in conjunction with those for the oxidation of H_2_ (group 1b [NiFe]-hydrogenases) and acetate (acetyl-CoA decarbonylase/synthase; *cooS*/*acsB*). In support of the high abundance of uncultured Desulfobacterales in the community analysis (Fig. 2a), some Desulfobacterales MAGs are affiliated with previously unreported families (Fig. 3; Table S10). The terminal oxidases encoded by the MAGs differ in a manner consistent with the contrasting distributions of Desulfobacterota families (Fig. 2a; Table S5); families significantly enriched in the least disturbed samples (e.g., Desulfosarcinaceae) encode cytochrome *bd* and *cbb*_3_ oxidases known to detoxify O_2_ in sulfate-reducing bacteria [88], whereas a family enriched in moderately disturbed samples (Desulfocapsaceae) also encodes a potentially growth-supporting cytochrome *aa*_3_ oxidase. Dissimilatory sulfite reductases were also encoded by two other phyla recently inferred to be sulfate reducers, Gemmatimonadota (family UBA6960) [89] and Bacteroidota (Ignavibacteriaceae) [90] (Fig. 3); both families appear to be highly metabolically flexible and may also mediate aerobic growth (Table S10). The capacity for aerobic and anaerobic sulfide oxidation was more widespread, with 96 MAGs from 29 different families encoding sulfide-quinone oxidoreductases, flavocytochrome *c* oxidoreductases, or reverse dissimilatory sulfite reductases (Table S10). All genes associated with sulfide and thiosulfate oxidation were most abundant at site C (*p* < 0.05) (Fig. 3), in line these differences are compatible with the increased sulfide availability at this site (Fig. 1b, c) and the enrichment of chemolithoautotrophic sulfide oxidizers such as Sulfurovaceae (Fig. 2a); the two *Sulfurovum* MAGs confirm these bacteria encode genes for aerobic sulfide oxidation (*sqr*), thiosulfate oxidation (*soxB*), and carbon fixation via the reverse tricarboxylic acid cycle (*aclB*).

Our results also add to growing evidence that inorganic nitrogen metabolism in permeable sediments depends on complex interspecies interactions. Genes associated with nitrification (*amoA*, *nxrA*, *hbsT*) were in low abundance and, in agreement with the geochemistry (Fig. 1d) and ANCOM (Fig. 2d) results, largely confined to the relatively aerated site A (Fig. 3a). In contrast, genes for denitrification and DNRA were abundant in the metagenomic short reads (Fig. 3a) and encoded by 99 of the 169 MAGs (Fig. 3b), suggesting oxidized nitrogen compounds are preferred electron acceptors in permeable sediments. Overall, the genes for denitrification were abundant across the metagenomic reads (av. 32% of community), whereas those associated with DNRA were significantly lower (av. 8.8%). However, the ratio of genes encoding denitrification-associated nitrite reductases (*nirS*, *nirK*) compared to DNRA-associated nitrate reductases (*nrfA*) exhibited a strong decrease relative to disturbance level (*R*^2^ = 0.85, *p* = 0.0005, linear regression) from the most disturbed sample (site A 0–2 cm, ratio 5.9) to least disturbed sample (site C 14–16 cm, ratio 2.0). Based on the MAGs (Fig. 3; Table S10), *nirS* and *nirK* genes were primarily associated with facultatively anaerobic habitat generalists such as Woeseiaceae, Flavobacteriaceae, and Rhodobacteraceae, whereas *nrfA* is encoded by few MAGs (Fig. 3), including relatively rare families Ignavibacteriaceae (Bacteroidota) and Pontiellaceae (Verrucomicrobiota) [91]. Also notable is the patchwork distribution of nitrate, nitrite, nitric oxide, and nitrous oxide reductases between families, with no MAGs (even those with >95% completeness) encoding complete denitrification pathways (Table S6). The ecophysiological advantages of such specialization in sediments that otherwise select for metabolic versatility remain unclear, but these observations are compatible with recent findings in permeable sediments and other systems [21, 25, 92].

### Fermentation and respiration are more coupled in less disturbed sites

**Fig. 4:**
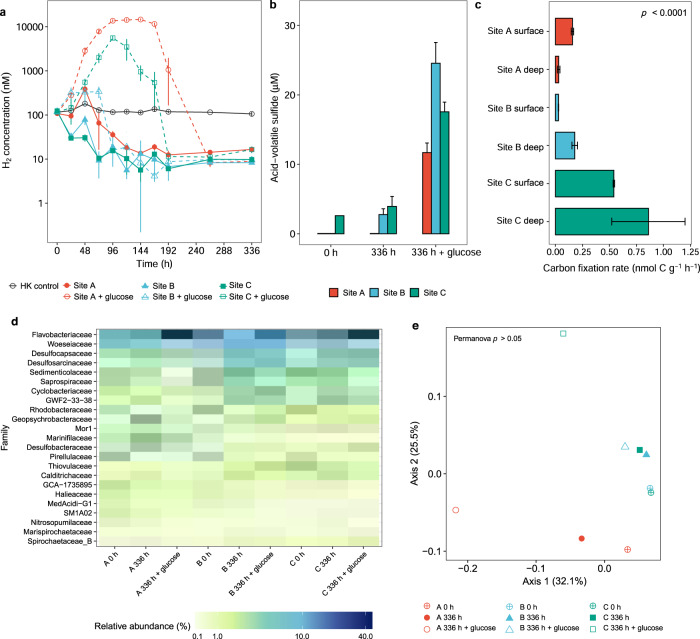
Differences in sulfate reduction and associated processes between sites. **a** Dissolved H_2_ concentrations in anoxic slurries amended with a headspace 100 ppmv H_2_. Samples either contained native organic carbon content or were spiked with 1 mM glucose. H_2_ production suggests hydrogenogenic fermentation, whereas H_2_ consumption indicates hydrogenotrophic sulfate reduction. Symbols show means and error bars show standard deviations from three independent replicates used per site. **b** Acid-volatile sulfide concentrations in sediments before and after anoxic incubations in slurries in the presence and absence of 1 mM glucose (one replicate per site for 0 h, three replicates per site for 336 h with error bars showing standard deviations). Free sulfide concentrations are shown in Table S11. **c** Comparison of rates of dark carbon fixation rates under oxic conditions and without electron donor spiking between surface and deep sands sampled from each site. Bars show means and error bars show standard deviations from three independent slurries. Significant differences were measured by one-way ANOVA. **d** Heatmap showing relative abundance of abundant microbial families, based on 16S rRNA gene amplicon sequencing, before and after anoxic incubations in the presence and absence of 1 mM glucose. **e** Beta diversity of samples from d based on weighted Unifrac analysis of 16S rRNA gene amplicon sequencing data and visualized by principal coordinates analysis (PCoA).

We performed a series of microcosm-based activities studies to validate the above metagenome-based inferences. First, we compared the coupling between fermentation and respiration processes in surface sediments by comparing rates of H_2_ production or consumption following a transition to anoxic conditions. For site A, fermentation rates initially exceeded respiration rates, resulting in a fourfold increase in H_2_ concentrations after 48 h (Fig. 4a; Table S11). In these sediments, net hydrogenotrophic respiration was observed only after prolonged anoxia, though sulfide levels remained below limits of detection (Table S11; Fig. 4b). In contrast, fermentation and respiration processes were tightly coupled in the less disturbed sites B and C, and hence H_2_ consumption was observed immediately following the onset of anoxia (Fig. 4a). Reflecting these differences, high levels of sulfide were detectable in sites B (free sulfide 1.2 µM, AVS 2.7 µM) and C (free sulfide 14.1 µM, AVS 3.9 µM) following the incubations (Table S11; Fig. 4b), suggesting efficient coupling of H_2_ oxidation to sulfate reduction. Based on 16S rRNA gene amplicon sequencing (Table S12), community composition of sediments from site A also diverged from those of sites B and C during the incubations (Fig. 4e). Notably, Desulfobacteraceae and Desulfocapsaceae grew to become the dominant sulfate-reducing bacteria in site A, compared to Desulfosarcinaceae in sites B and C (Fig. 4d).

Addition of the fermentable carbon source glucose accentuated differences between the three sites. H_2_ accumulated to mixing ratios above 1% in site A, though unexpectedly also reached high levels in site C and remained low for the stably coupled site B. Strong coupling between fermentation and respiration was only observed after four and eight days of prolonged anoxia for sites C and A respectively (Fig. 4a; Table S11). AVS measurements confirmed all sites eventually mediated high rates of hydrogenotrophic sulfate reduction (Fig. 4b). Community composition differences provide some rationale for these divergent responses. Strong community shifts occurred following glucose spiking for sites A and C (Fig. 4d, e), including rapid growth of facultative fermenters (Flavobacteriaceae harboring group 3 NiFe-hydrogenases; Table S12) and obligate fermenters (e.g., Spirochaetaceae likely encoding FeFe-hydrogenases [93]) relative to the three above-mentioned hydrogenotrophic sulfate-reducing families (encoding group 1 NiFe-hydrogenases; Table S12). In contrast, a much milder response was observed for site B (Fig. 4d, e). Overall, these findings extend previous observations that hydrodynamic disturbance causes sediments to become uncoupled, by selecting for facultatively anaerobic fermentative bacteria and excluding obligately anaerobic sulfate reducers [21, 23]. Moreover, they clearly confirm that differences in hydrodynamic gradients impact not only community structure, but also biogeochemical reactions.

In addition, we tested the inferences from community and functional profiling that sulfide-oxidizing chemolithoautotrophic bacteria (e.g., Sulfurovaceae) are enriched in site C (Figs. 2 & 3) by measuring rates of dark carbon fixation and assimilation under oxic conditions. Consistent with predictions, rates in site C were on average seven-fold higher compared to sites B and C (*p* < 0.0001, one-way ANOVA) (Fig. 4c; Table S11). Rates were minimally affected by supplementation with additional sulfide or ammonium (Fig. S7), suggesting the high levels of sulfide and potentially other electron donors already present in site C drive most fixation (Fig. 1b, c), though some sulfide-dependent stimulation of carbon fixation was observed in the sulfide-depleted surface sands of sites A and B (Fig. S7). Overall, these assays provide supporting evidence that sulfide accumulation due to activities of sulfate-reducing bacteria in less disturbed sediments stimulates chemolithoautotrophic growth when oxygen becomes available. However, tracing studies (e.g., stable isotope probing; SIP) would be required to confirm which microorganisms are differentially active between the sites.

### Ratios of denitrification to dissimilatory nitrate reduction to ammonium are lower in less disturbed sites

**Fig. 5:**
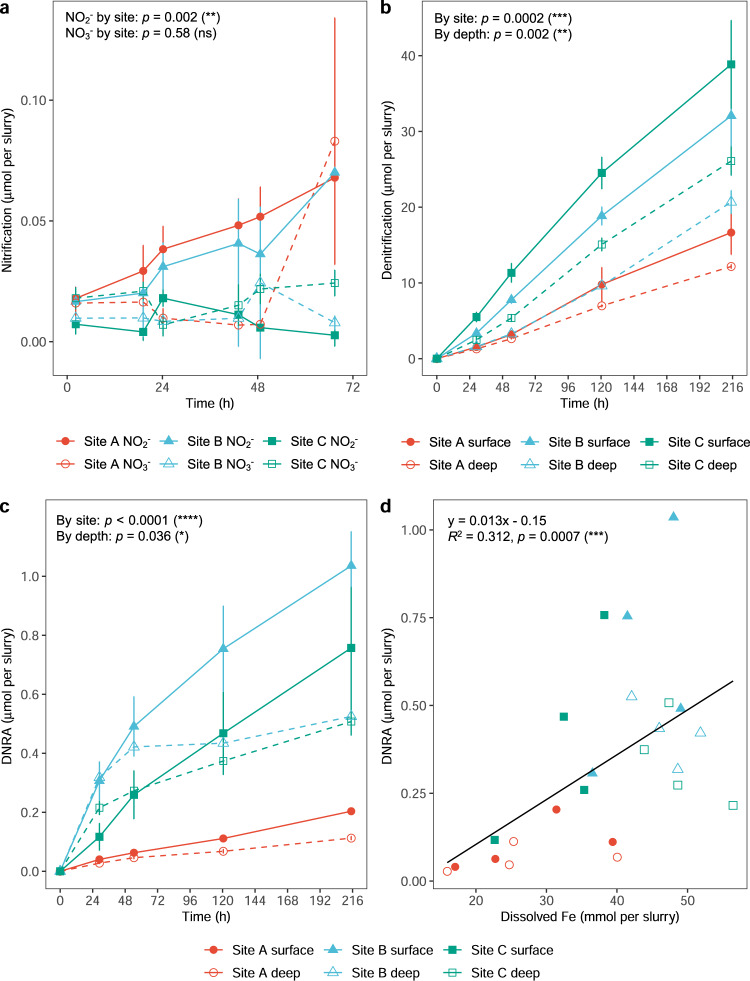
Differences in nitrogen-cycling processes between sites. **a** Cumulative nitrification in oxic slurries amended with 50 µM NH_4_Cl, as measured by NO_2_^−^ and NO_3_^−^ production. **b** Cumulative denitrification in anoxic slurries amended with 1 mM Na^15^NO_3_, as measured by ^15^N-N_2_ production. **c** Cumulative dissimilatory nitrate reduction to ammonium (DNRA) in anoxic slurries amended with 1 mM Na^15^NO_3_, as measured by ^15^N-NH_4_^+^ production. Results are shown for sediments of 0–5 cm depth were used for nitrification measurements. Results are shown for sediments of both 0–5 cm (shallow) and 20–25 cm (deep) depth for denitrification and DNRA measurements. Symbols show means and error bars show standard deviations from three independent slurries. ANCOVA tests of linear regressions were used to test significant differences by site (**a**, **b**, **c**) and by depth (**b**, **c**). **d** Correlation of DNRA rate with dissolved iron content based on linear regression analysis.

Finally, we measured rates of nitrification, denitrification, and DNRA rates between sites. As predicted from the metagenomic analysis (Fig. 3) and in support of some previous findings [29, 94], nitrification rates were slower than denitrification and DNRA rates (Fig. 5; Table S13). Nitrification rates in oxic microcosms containing surface sands were highest for site A and negligible for site C (*p* = 0.002, ANCOVA; Fig. 5a) in agreement with differences in the relative abundance of ammonia-oxidizing microorganisms (Nitrosopumilaceae; Fig. 2a) and genes (*amoA*; Fig. 3). NH_4_^+^ was converted primarily to NO_2_^−^ in sites A and B, and some conversion to NO_3_^−^ was observed after prolonged incubation at site A (Table S13). This suggests ammonia oxidation exceeds nitrite oxidation. Following oxic-anoxic transitions, it is likely that much of the nitrite produced would be reduced through denitrification, in line with previous reports of nitrifier denitrification in permeable sediments [30, 94]. Ammonium supplementation did not result in a detectable enrichment in dark carbon fixation above background levels (Fig. S7).

Rates of denitrification and DNRA were measured by comparing ^15^N_2_ and ^15^NH_4_^+^ production in anoxic microcosms spiked with Na^15^NO_3_. As also predicted from the metagenomic analyses (Fig. 3), denitrification occurred at rapid rates throughout the 216 h time-course (Fig. 4c). Cumulative denitrification rates significantly varied by site (*p* = 0.0002, ANCOVA), with highest activities at sites B and C and lowest activities in site A, in line with expectations based on their relative disturbance levels. Rates were also higher in surface compared to deeper sediments (*p* = 0.002, ANCOVA), likely reflecting their higher labile carbon and microbial abundance (Fig. 4c; Table S13). In contrast, DNRA rates were one to two orders of magnitude lower than denitrification (Fig. 4d) and again occurred at higher rates at less disturbed sites (*p* < 0.0001, ANCOVA) and deeper sediment depths (*p* = 0.036, ANCOVA) Table S13).

Reflecting shifts in the ratios of denitrification (*nirK*, *nirS*) to DNRA (*nrfA*) genes (Fig. 3), the ratio of denitrification to DNRA was approximately twofold lower for the less disturbed sites (*p* < 0.0001, ANCOVA) and increased during the time-courses (Fig. S8; Table S13). It should be noted that these ratios may be skewed given nitrate concentrations used in these assays are much higher than typical in situ concentrations, though this is unlikely to affect comparisons between sites. DNRA was correlated with dissolved Fe concentrations (*p* = 0.0007, ANCOVA), suggesting that it may be mediated by lithotrophs in these sediments (Fig. 5d). No accumulation of sulfide was detected in these slurries. This may reflect two factors. First, given nitrate is a stronger electron acceptor than sulfate, those sulfate reducers that harbor nitrate reduction genes (Fig. 3) may preferentially use nitrate when it is abundant. Second, in line with the widespread co-occurrence of sulfide oxidation and nitrate reduction genes (Fig. 3), any sulfide produced by sulfate reducers may be immediate reoxidised by sulfide-oxidizing nitrate reducers. Together with the sulfate reduction assays (Fig. 4), these findings further support the third hypothesis that respiration processes associated with obligate anaerobes are more active in less disturbed sediments.

## Discussion

Here we provide multifaceted evidence that hydrodynamic disturbance is a major driver of microbial composition and biogeochemical pathways in permeable sediments. In support of our three original hypotheses, we show that geochemical stratification, microbial specialism, and biogeochemical coupling increases in less disturbed sites. Thus, as hydrodynamic forcing decreases, conditions sufficiently stabilize for obligately anaerobic specialists to increasingly outcompete the facultatively anaerobic generalists that dominate at more disturbed sites. For example, we provide multifaceted evidence that the presence and activity of sulfate-reducing bacteria are differentiated by disturbance level: (i) sulfide concentrations are higher in less disturbed sediments, (ii) relative abundance of sulfate-reducing bacteria and their marker genes is inversely correlated with sediment disturbance level, (iii) MAGs of known and novel families of sulfate-reducing bacteria were recovered from sediments with low and intermediate disturbance, and (iv) anoxic carbon mineralization processes are strongly coupled to sulfate reduction only in sites with low or intermediate disturbance. Such findings confirm previous reports that sediment disturbance and permeability influences community composition [18, 21], and supports our previously published conceptual frameworks on the distributions and traits of habitat generalists versus specialists in these environments [21–23, 32].

Some findings were nevertheless contrary to our original expectations. Most notably, though we observed strong differentiation between the mixing and sulfidic zones, we observed that depth stratification in microbial composition and activities was otherwise modest even in sites with low disturbance. Many of the families and even some individual ASVs were shared across all samples, albeit at varying relative abundances. Also in contrast to highly stratified cohesive sediments, common biogeochemical activities were observed in samples from different sites and depths, albeit at distinct rates. One explanation is that, unlike cohesive sediments, there is still considerable mixing of compounds and microorganisms through porewater advection even in less frequently disrupted sands [2]. Indeed, single sand grains harbor diverse and abundant communities that can be readily dispersed [19]. These observations also likely reflect that many of the bacteria present can grow or survive despite changes in resource availability and other physicochemical factors. In agreement with previous work [21–23, 25], we observed taxa such as Flavobacteriaceae, Woeseiaceae, and Rhodobacteraceae possess sufficient metabolic flexibility to grow in both light oxic and dark anoxic conditions, explaining their abundance in even the least disturbed sands. However, even some apparent anaerobes appear to possess considerable flexibility. For example, we predict that the Desulfocapsaceae present across all samples and enriched in sediments with intermediate disturbance are either highly aerotolerant or potentially even capable aerobic growth, especially given the MAG-level analysis shows they encode cytochrome *aa*_3_ oxidases.

In turn, our analyses suggest that disturbance operates as a continuous variable in permeable sediments. Reflecting this, we observed strong linear correlations between sediment disturbance level with sulfide and ammonium concentrations, relative abundance of microbial families as inferred from 16S rRNA gene amplicon sequencing, and abundance of metabolic genes as inferred from metagenomic short reads. The most abundant families were present in all samples, though the habitat generalists increased in relative abundance with disturbance level and the relative anaerobic specialists showed the opposite trend, in a manner compatible with their metabolic capabilities. Only a few families, for example the key aerobic specialist Nitrosopumilaceae [21] or the sulfide-oxidizing chemolithoautotroph Sulfurovaceae [81], displayed contrary trends. Assuming high levels of dispersal in these ecosystems, we predict that disturbance level interacts with other deterministic factors to shape growth and survival dynamics of each taxon, thereby controlling their relative abundance in a potentially predictable way. However, it should be noted that disturbance level did not explain the abundance and diversity of the overall microbial community, suggesting other as-yet-unrecognized overarching controls also operate.

By combining genome-resolved metagenomics with microcosm-based biogeochemical assays, we additionally developed understanding of the mediators of biogeochemical processes in permeable sediments. Collectively, the data suggests that fermentation is the dominant process of anoxic carbon mineralization in highly disturbed sediments as previously predicted [23, 28], but that the classical suite of heterotrophic mineralization reactions (e.g., denitrification, DNRA, and sulfate reduction) occur at higher rates in less disturbed sediments. These differences likely reflect the complex effects of hydrodynamic disturbance on the relative distributions, gene expression, and enzymatic activities of habitat generalists and anaerobic specialists. Denitrification is a more active process than DNRA and appears to be mediated by more community members, in support of earlier findings [27, 74]. However, given the MAGs only encode partial denitrification pathways, this process is likely to depend on extensive metabolic interactions between species for unclear reasons. It is proposed that organism specialization for each step in the denitrification pathway is more thermodynamically efficient than one organism mediating the multiple steps [95]. In combination, the stepwise pathways and diverse mediators of nitrification, denitrification, and DNRA suggest complex ecological interactions control nitrogen cycling in permeable sediments. Our findings also provide a deeper understanding of the diversity and capabilities of sulfate-reducing bacteria present in these environments; marker genes for sulfate reduction were detected in differentially distributed lineages of Desulfobacterota, together with surprisingly Gemmatimonadota and Bacteroidota, suggesting complex controls on sulfate reduction. Low levels of acetogenic and methanogenic archaea were also observed in the least disturbed sediments, though it remains to be confirmed if they are active. Our analyses also highlight the key roles of lithoheterotrophy and lithoautotrophy in permeable sediment function, as suggested by the wide distribution of genes for sulfide, hydrogen, and carbon monoxide oxidation and the diverse pathways for carbon fixation.

Future studies should focus on integrating culture-based and culture-independent approaches to gain a deeper perspective on the ecophysiology of the major taxa in these ecosystems. We recommend targeted tracing studies (e.g., DNA-SIP) and metatranscriptomics to better link biogeochemical activities to mediators; similar approaches have previously helped to confirm taxa such as Woeseiaceae, Desulfocapsaceae, and uncultured Desulfobacterales are key mediators of sulfur and carbon cycling in permeable sediments [22, 24, 32, 87, 96]. In addition, more extensive cultivation-based studies are needed to validate metabolic predictions, for example to confirm the metabolic versatility of Woeseiaceae, test the potential for aerobic growth of Desulfocapsaceae, and extend the capacity for sulfate reduction and DNRA to other phyla. Such approaches are also important to understand physiological responses to changes in resource availability (e.g., oxic-anoxic transitions). Further work is needed to understand the dynamics and drivers of temporal turnover in permeable sediments, including seasonality, and to what extent this is affected by disturbance level. Wider spatial and temporal sampling is also important to understand what drives the unexplained differences in microbial abundance and biodiversity between samples, and resolve which other factors interact with disturbance level to control microbial composition and biogeochemical activities.

## Supplementary information

Supplementary information

Supplementary Table S1

Supplementary Table S2

Supplementary Table S3

Supplementary Table S4

Supplementary Table S5

Supplementary Table S6

Supplementary Table S7

Supplementary Table S8

Supplementary Table S9

Supplementary Table S10

Supplementary Table S11

Supplementary Table S12

Supplementary Table S13

## Data Availability

All amplicon sequencing data, raw metagenomes, and metagenome-assembled genomes will be deposited to the NCBI Sequence Read Archive under the BioProject accession number PRJNA623061.
