# Supplementary material for: Hydrodynamic disturbance controls microbial community assembly and biogeochemical processes in coastal sediments
Source: ISME J. 2021 Sep 28;16(3):750–63. doi: 10.1038/s41396-021-01111-9 (PMC8857189; doi:10.1038/s41396-021-01111-9)

**Supplementary information**

**Table S1 (xlsx).** Raw data on mixing layer depth, sediment grain size, geochemical measurements, and microbial abundance for each sediment core.

**Table S2 (xlsx).** ASV table showing community composition of bacterial and archaeal communities in each sediment core slice based on 16S rRNA gene amplicon sequencing.

**Table S3 (xlsx).** Reads removed during DADA2 analysis of 16S rRNA gene amplicon sequencing data.

**Table S4 (xlsx).** Alpha diversity of bacterial and archaeal communities in each sediment core slice.

**Table S5 (xlsx).** Analysis of relative abundance of microbial taxa based on linear regression, ANOVA, and ANCOM analyses.

**Table S6 (xlsx).** Sequencing and assembly details of the nine shotgun metagenomes.

**Table S7 (xlsx).** Nearest taxonomic unit (NTU) summary of microbial community composition based on PhyloFlash.

**Table S8 (xlsx).** Taxonomy summary of microbial community composition based on PhyloFlash.

**Table S9 (xlsx).** Abundance and linear regression analysis of metabolic marker genes in sediment metagenome short reads.

**Table S10 (xlsx)**. Summary of taxonomy, quality statistics, coverage, and genetic capabilities of the 169 metagenome-assembled genomes.

**Table S11 (xlsx).** Measurements of H_2_ concentrations, sulfide concentrations, and dark carbon fixation in slurries.

**Table S12 (xlsx).** ASV table showing microbial community in slurry experiment before and after 14 days of anoxia with and without glucose spiking based on 16S rRNA gene amplicon sequencing.

**Table S13 (xlsx).** Measurements of nitrification, denitrification, and dissimilatory nitrate reduction to ammonium rates based on ^15^N analysis and measurements of soluble iron.

**Figure S1.** Location of the three sampling sites in Port Philip Bay in relation to St. Kilda Breakwater. North is shown by the arrow on the top-right. Inset shows wind rose at 3 hourly intervals over five years from 01 Jan 2015 to 31 Dec 2019. Map modified from igismap.com under Open Data Commons Open Database License (ODbL) using QGIS. Wind data supplied by the Australian Bureau of Meteorology from "St Kilda Harbour - RMYS station number 086220", located on the pier adjacent to the St. Kilda breakwater.

**
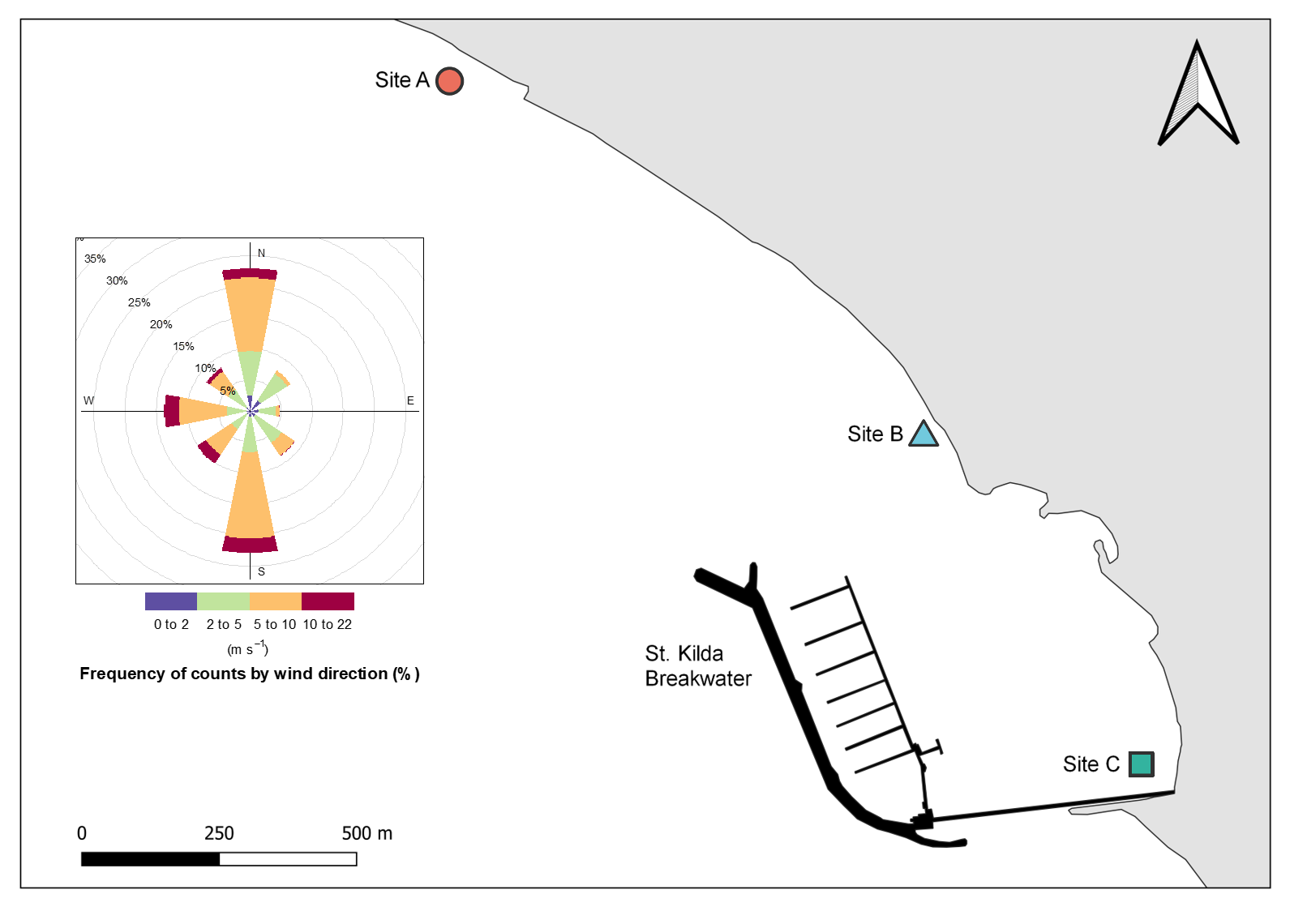
**

**Figure S2.** Linear regression analysis comparing geochemical measurements, microbial abundance, and microbial diversity versus sample disturbance level (measured by average distance from sulfidic layer in cm). Lines of best fit are shown where *p* < 0.05.


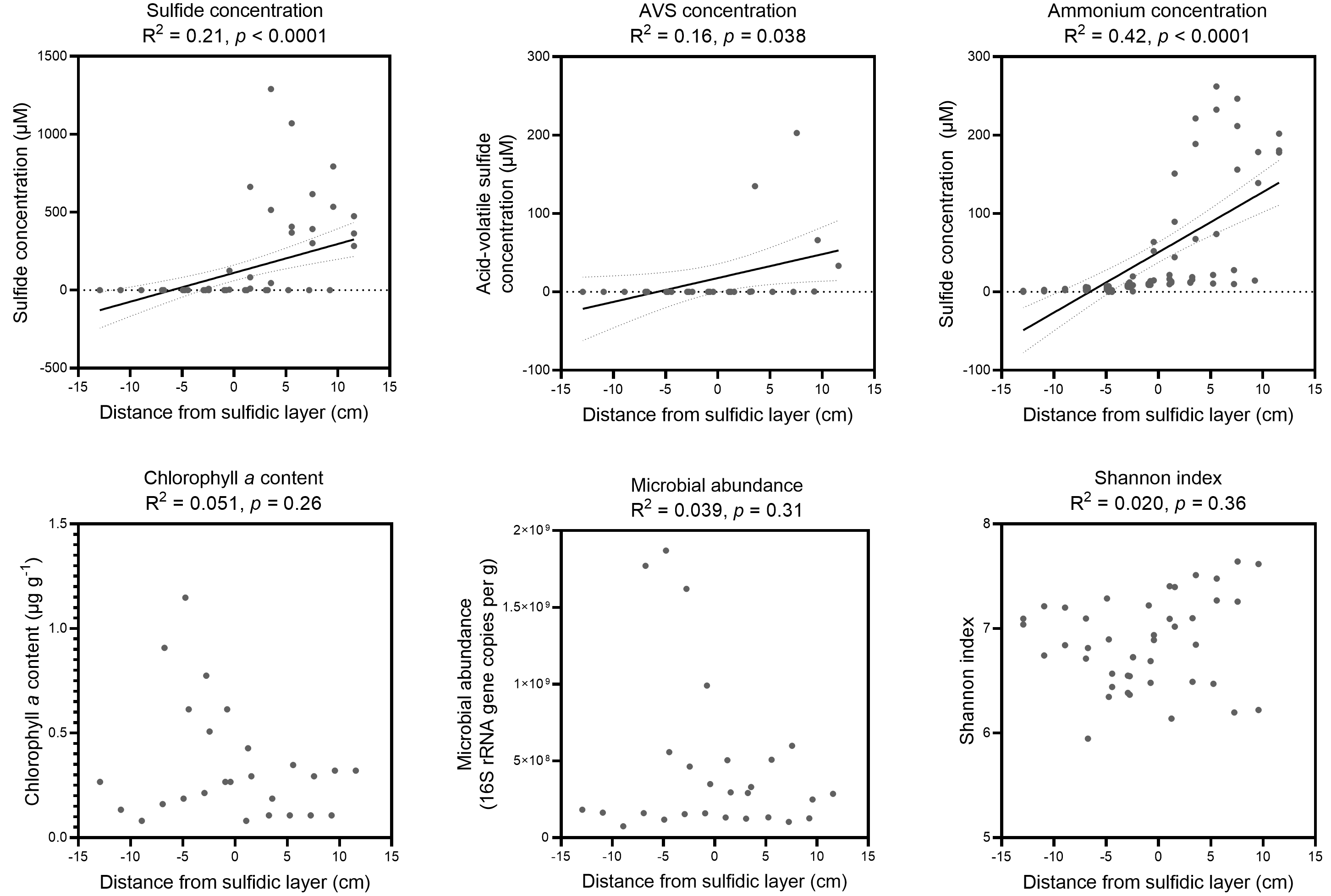


**Figure S3.** Relative abundance of key bacterial and archaeal taxa between sites and depths. Shown are the relative abundance of the 35 most abundant families **(a, c)** and the 10 most differentiated families **(b, d)**. Bars show means and error bars show standard deviations. Statistical significance is based on one-way ANOVA. * *p* < 0.05, ** *p* < 0.01, *** < 0.001, **** *p* < 0.0001, ns not significant.

**
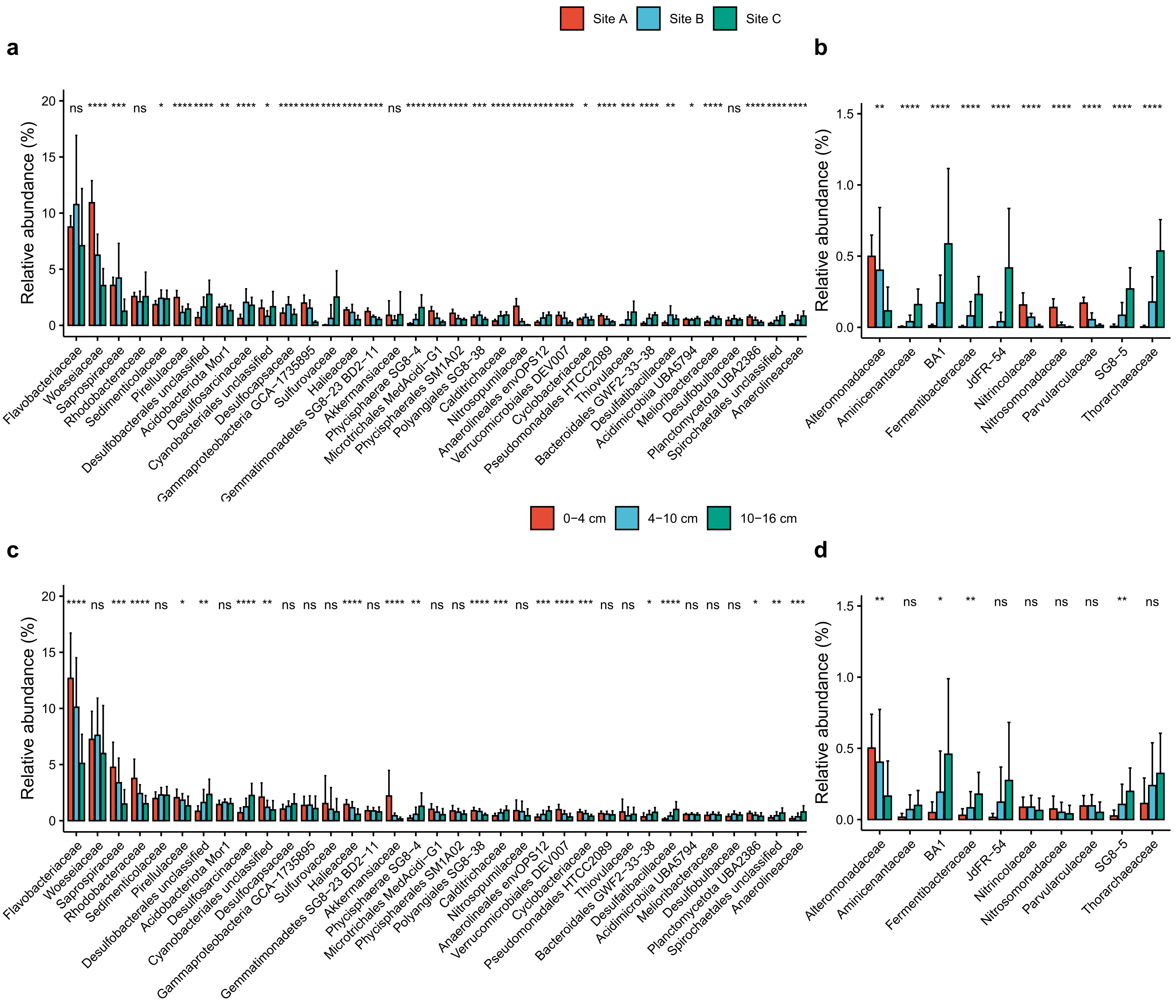
**

**Figure S4.** Linear regression analysis comparing microbial relative abundance versus sample disturbance level (measured by average distance from sulfidic layer in cm). Graphs are shown for the 35 most abundant named bacterial and archaeal families depicted in Figure 2a (full details in **Table S5**). Lines of best fit are shown where *p* < 0.05.

**
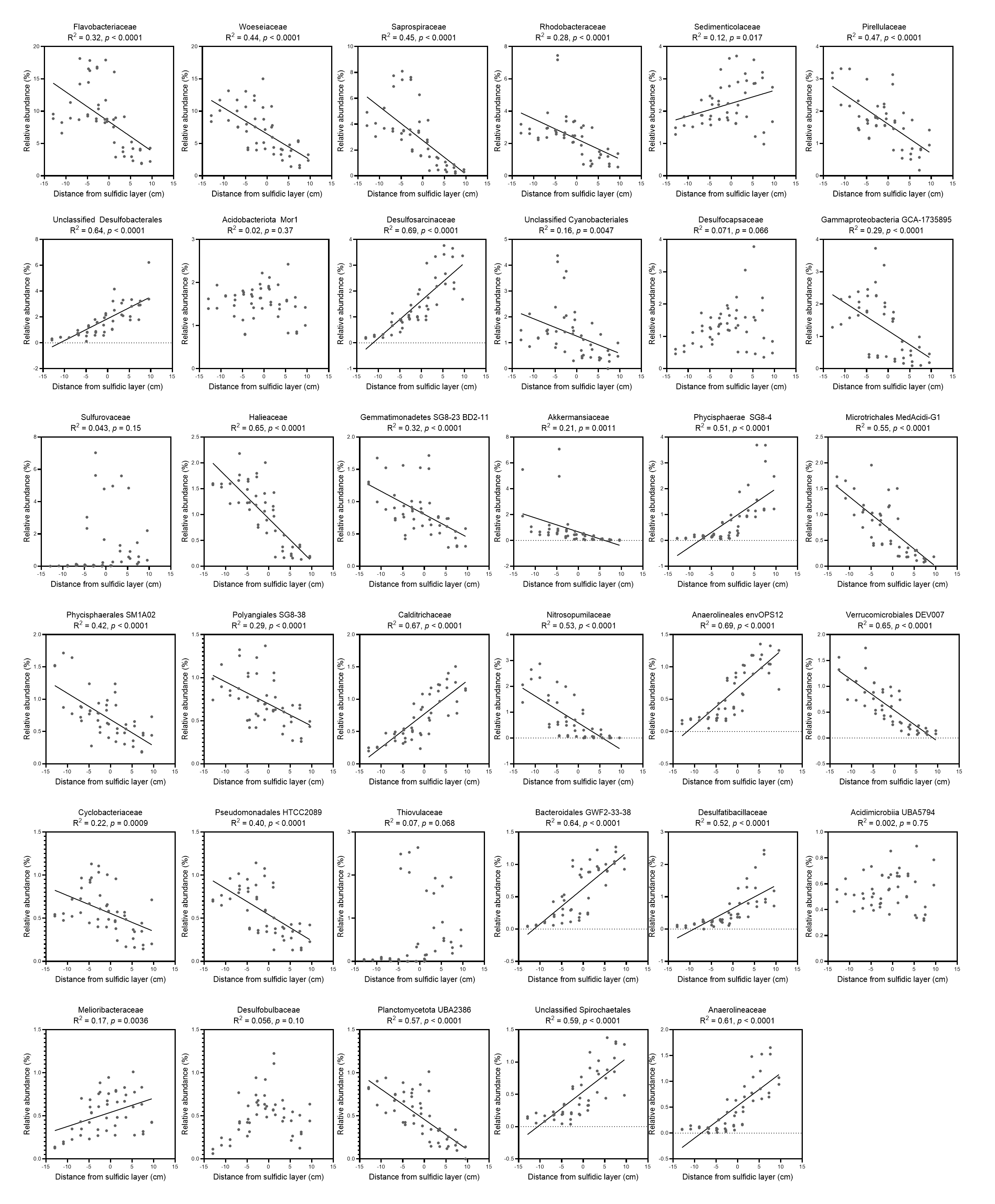
**

**Figure S5.** Taxonomic affiliations of the rRNA small subunit genes (SSU) retrieved by PhyloFlash. **(a)** Relative proportion of bacterial, archaeal, and organellar 16S rRNA gene and eukaryotic 18S rRNA gene reads. **(b)** Composition of eukaryotic community composition based on affiliation of 18S rRNA gene reads.

**
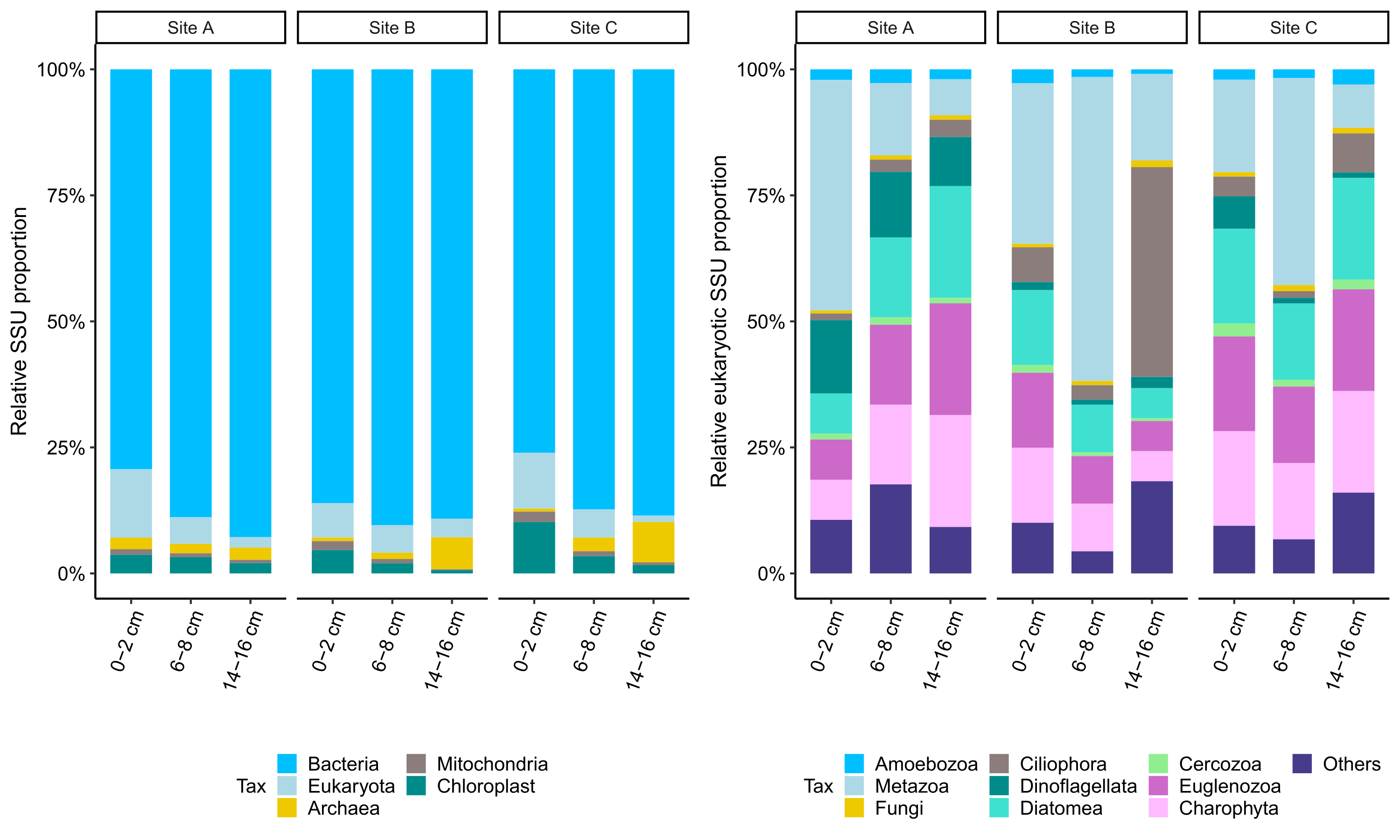
**

**Figure S6.** Linear regression analysis comparing metabolic marker gene abundance versus sample disturbance level (measured by average distance from sulfidic layer in cm). Graphs are shown for the 20 selected marker genes (full details in **Table S9**). Lines of best fit are shown where *p* < 0.05.


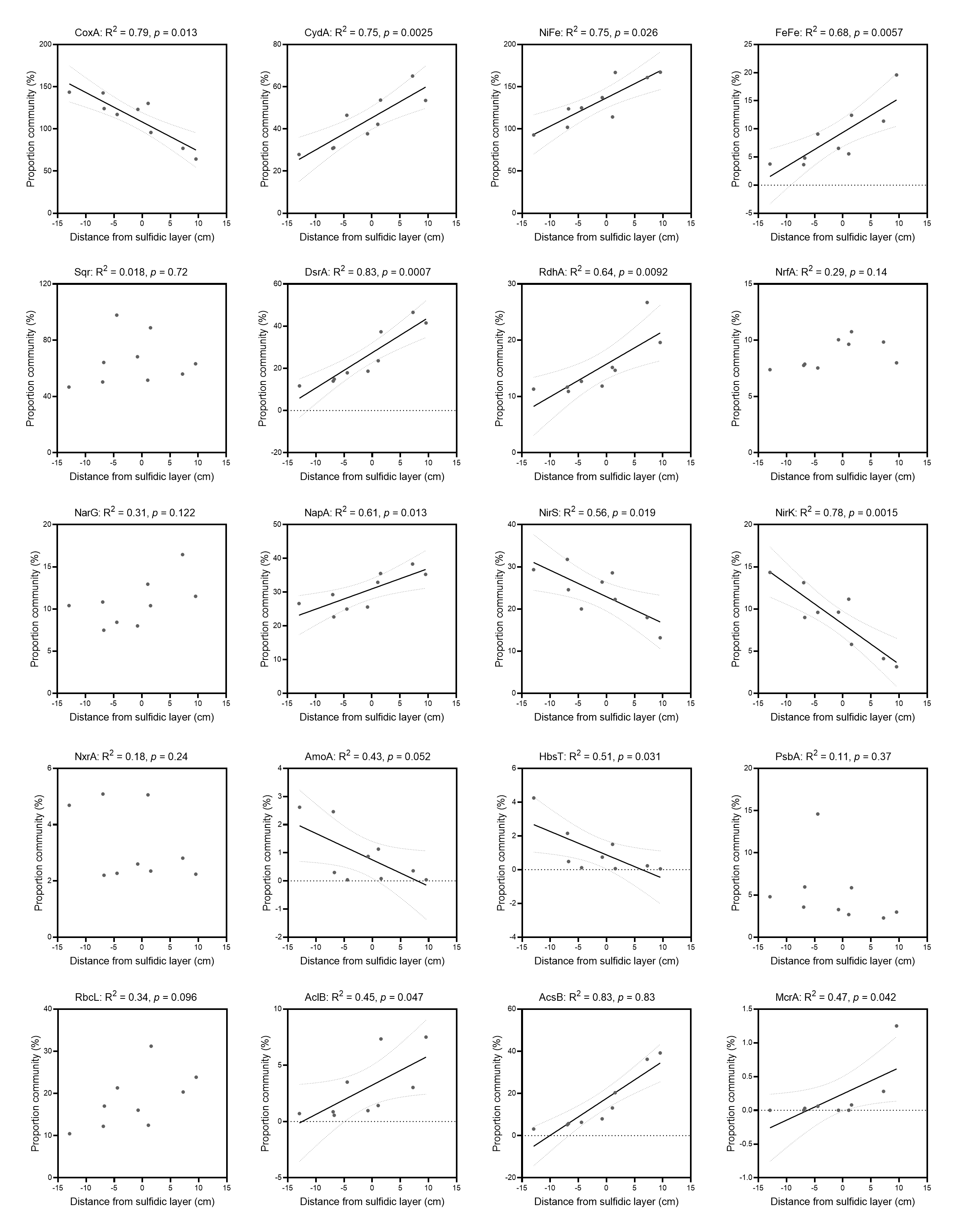


**Figure S7.** Rates of dark carbon fixation between the sites. Sands were incubated in serum vials with native electron donors (unamended) or following the additions of 200 µM sodium sulfide (Na_2_S.9H_2_O) or 200 µM ammonium chloride (NH_4_Cl). Bars show means and error bars show standard deviations from three independent slurries. Significant differences were measured by one-way ANOVA.

**
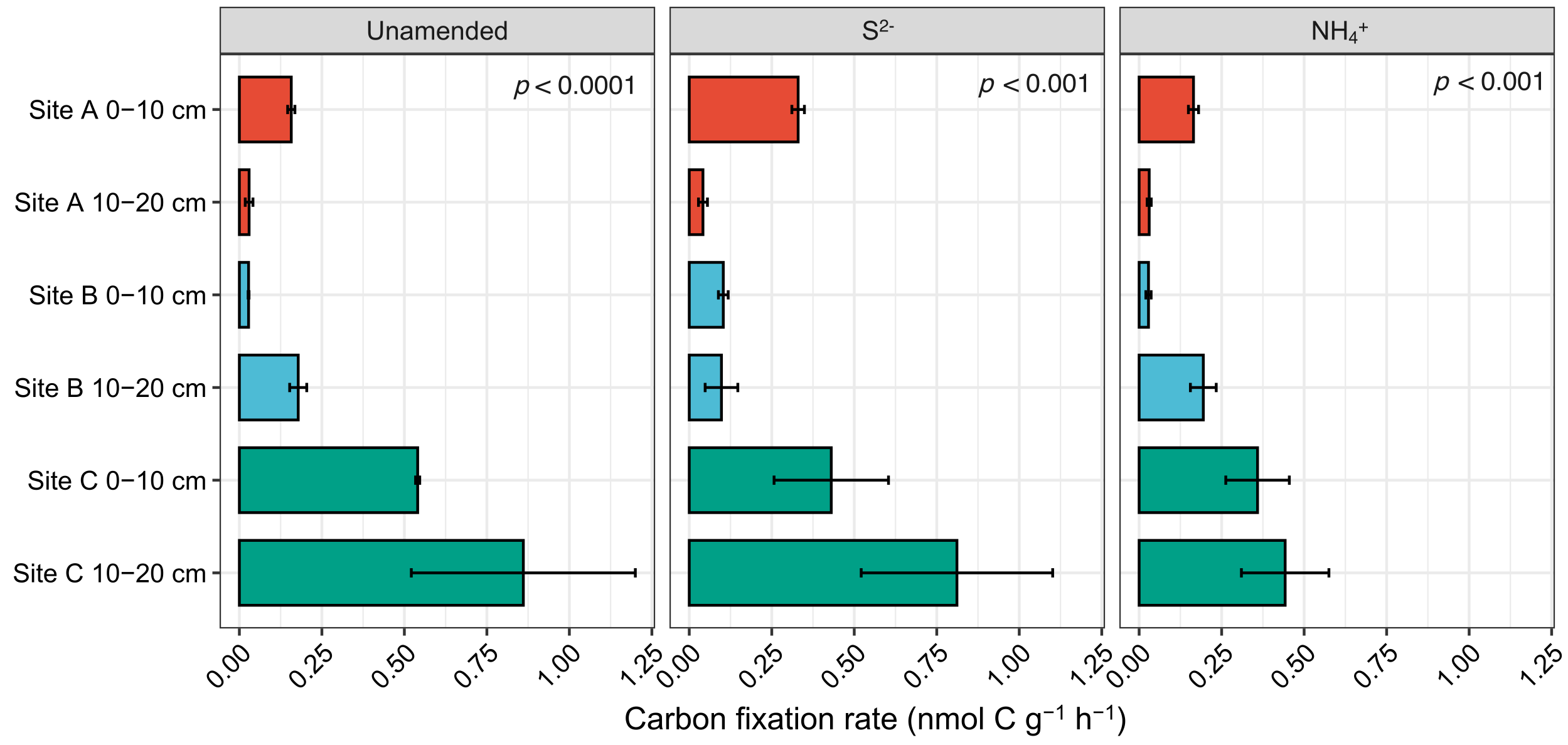
**

**Figure S8.** Ratio of denitrification to DNRA in anoxic slurries incubated with 1 mM Na^15^NO_3_. Results are compared between the three sites for shallow (0-5 cm depth) and deep (20-25 cm depth) sediments.


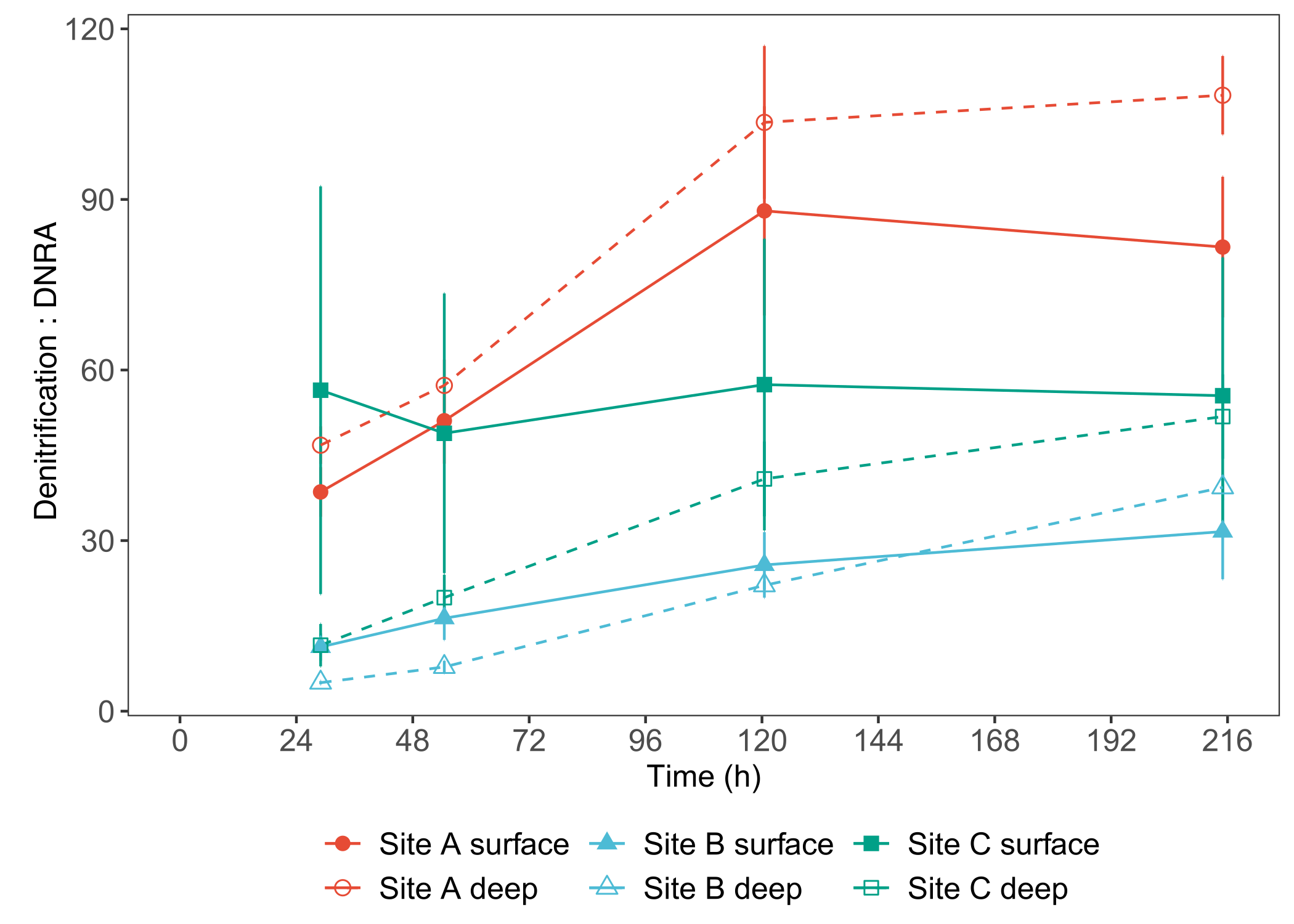

Supplement: Supplementary file 1 — Supplementary information [file 41396_2021_1111_MOESM1_ESM.docx]
